# Supplementary material for: The ZZ-type zinc finger of ZZZ3 modulates the ATAC complex-mediated histone acetylation and gene activation
Source: Nat Commun. 2018 Sep 14;9:3759. doi: 10.1038/s41467-018-06247-5 (PMC6138639; doi:10.1038/s41467-018-06247-5)
Supplement: Supplementary file 1 — Supplementary Information [file 41467_2018_6247_MOESM1_ESM.pdf]

Supplementary Information

The ZZ-type zinc finger of ZZZ3 modulates the ATAC complex-mediated histone acetylation and gene activation

Mi et al.

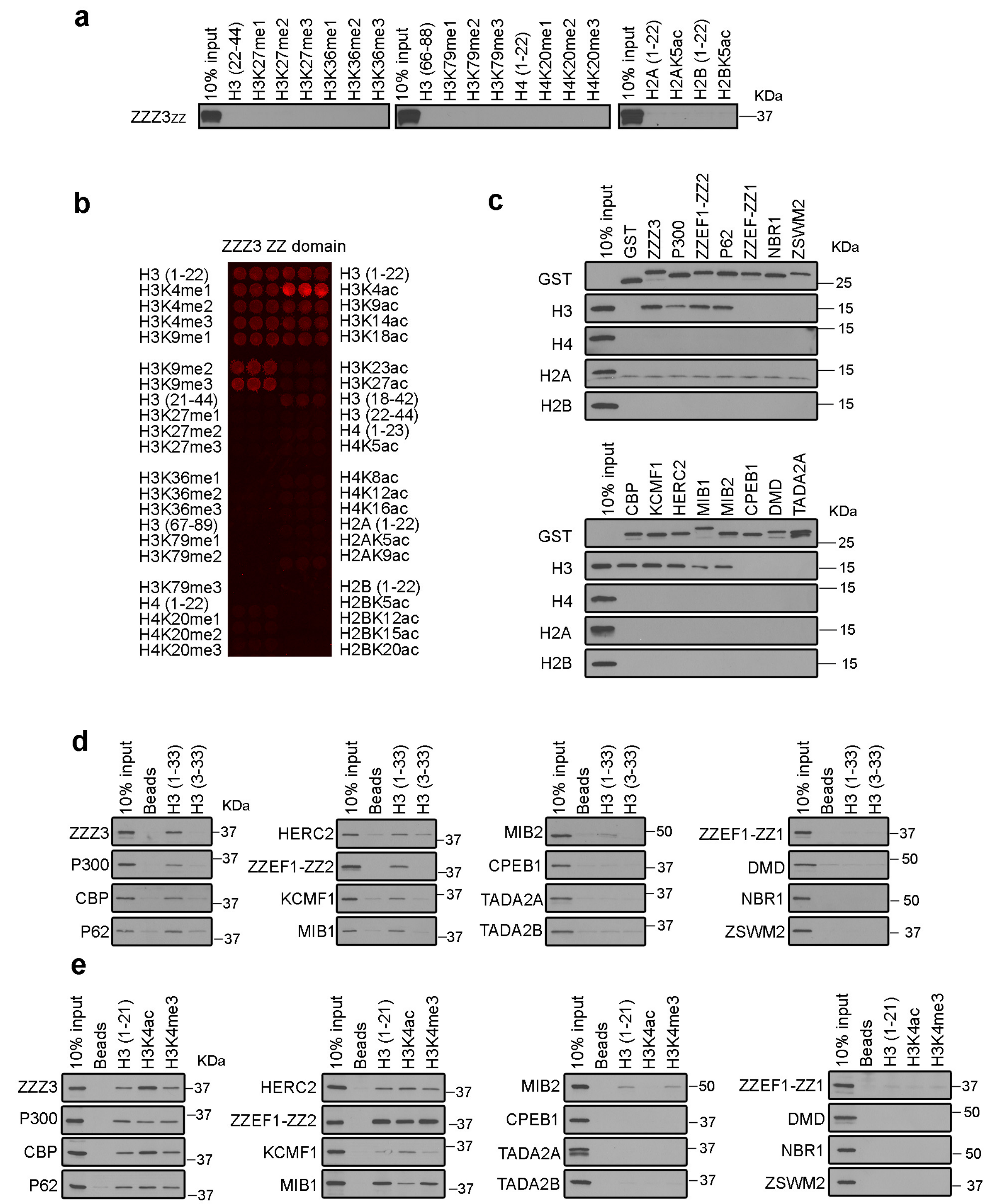

Supplementary Figure 1. Recognition of histone H3 is a common feature of human nuclear ZZ domains. a, Western blot analysis of histone peptide pulldowns of GST-ZZZ3 ZZ domain. **b,** Histone peptide microarray probed with GST-ZZZ3 ZZ domain. Each peptide has three replicates. **c,** Western blot analysis of GST pulldowns of the indicated human nuclear ZZ domains with calf thymus histones. **d** and **e,** Western blot analysis of histone peptide pulldowns of the 16 nuclear ZZ domains with the indicated histone peptides.

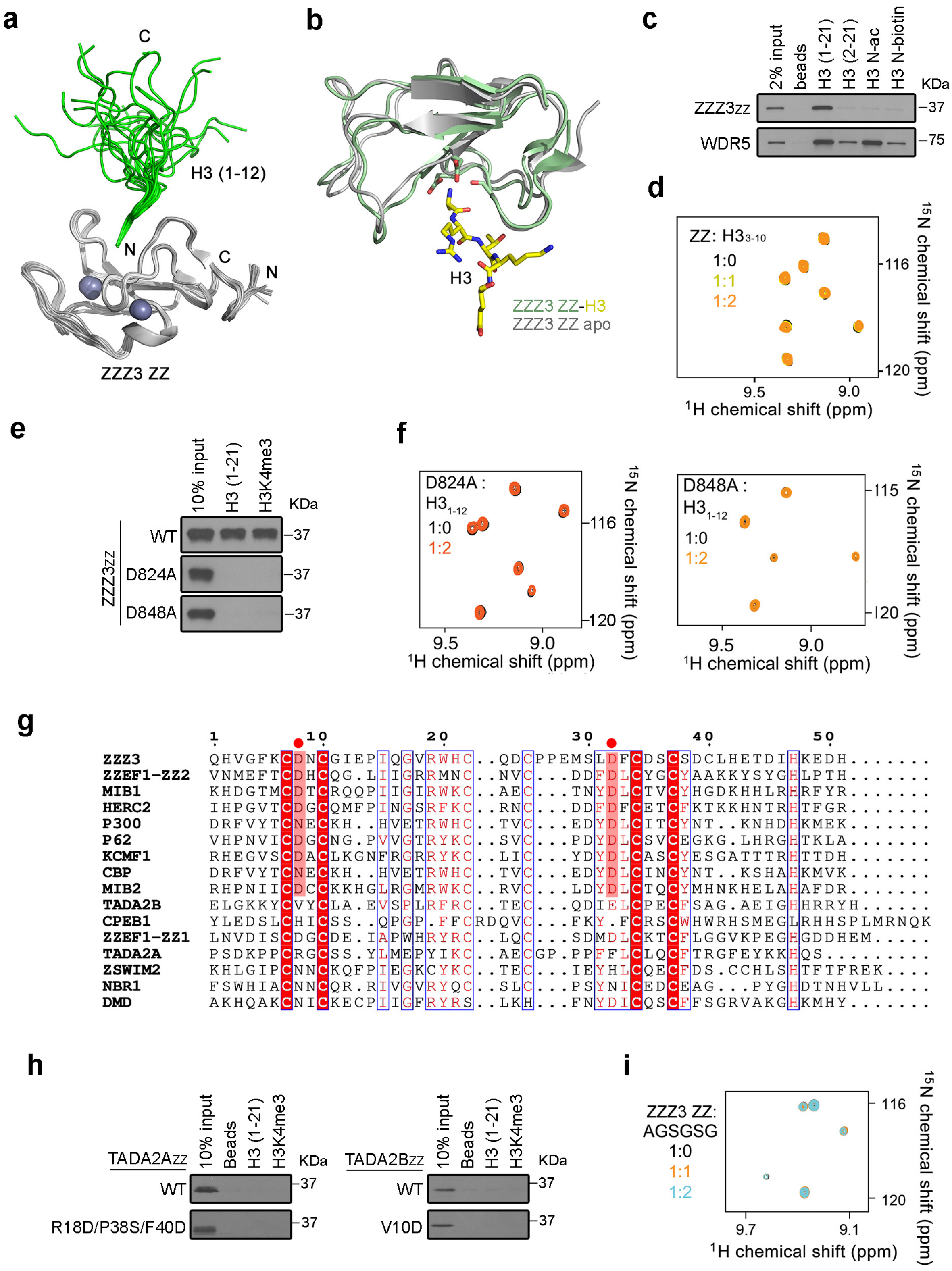

Supplementary Figure 2. Recognition of histone H3 tail by the ZZZ3 ZZ domain.

a, Superimposition of 20 final NMR structures of the ZZZ3 $ZZ_{816-874}$ bound to the H3 (1-12) peptide (green). **b,** Structural superimposition of apo- (PDB ID: 2FC7) and H3-bound ZZZ3 ZZ. **c**, Western blot analysis of indicated histone peptide pulldowns of GST-ZZZ3 ZZ domain. **d**, Superimposed $^{1}H,^{15}N$ HSQC spectra of the ZZZ3 $ZZ_{816-874}$ collected upon titration with the H3 (3-10) peptide. Spectra are color coded according to the protein-to-peptide molar ratio. **e**, Western blot analysis of histone peptide pulldowns of wild type (WT) ZZZ3 ZZ and the indicated point mutants. **f,** Superimposed $^{1}H,^{15}N$ HSQC spectra of the ZZZ3 $ZZ_{816-874}$ D824A and D848A mutants collected upon titration with the H3 (1-12) peptide. Spectra are color coded according to the protein-to-peptide molar ratio. **g**, Sequence alignment of human ZZ domains. Identical residues are shaded in red. Conserved residues are indicated in red and framed by blue line. Red dots, the conserved Ala-binding residues. **h,** Western blot analysis of histone peptide pulldowns of wild type (WT) TADA2A ZZ and TADA2B ZZ, and the indicated point mutants. **i,** Superimposed $^{1}H,^{15}N$ HSQC spectra of the ZZZ3 ZZ collected upon titration with the AGSGSG peptide. Spectra are color coded according to the protein-to-peptide molar ratio.

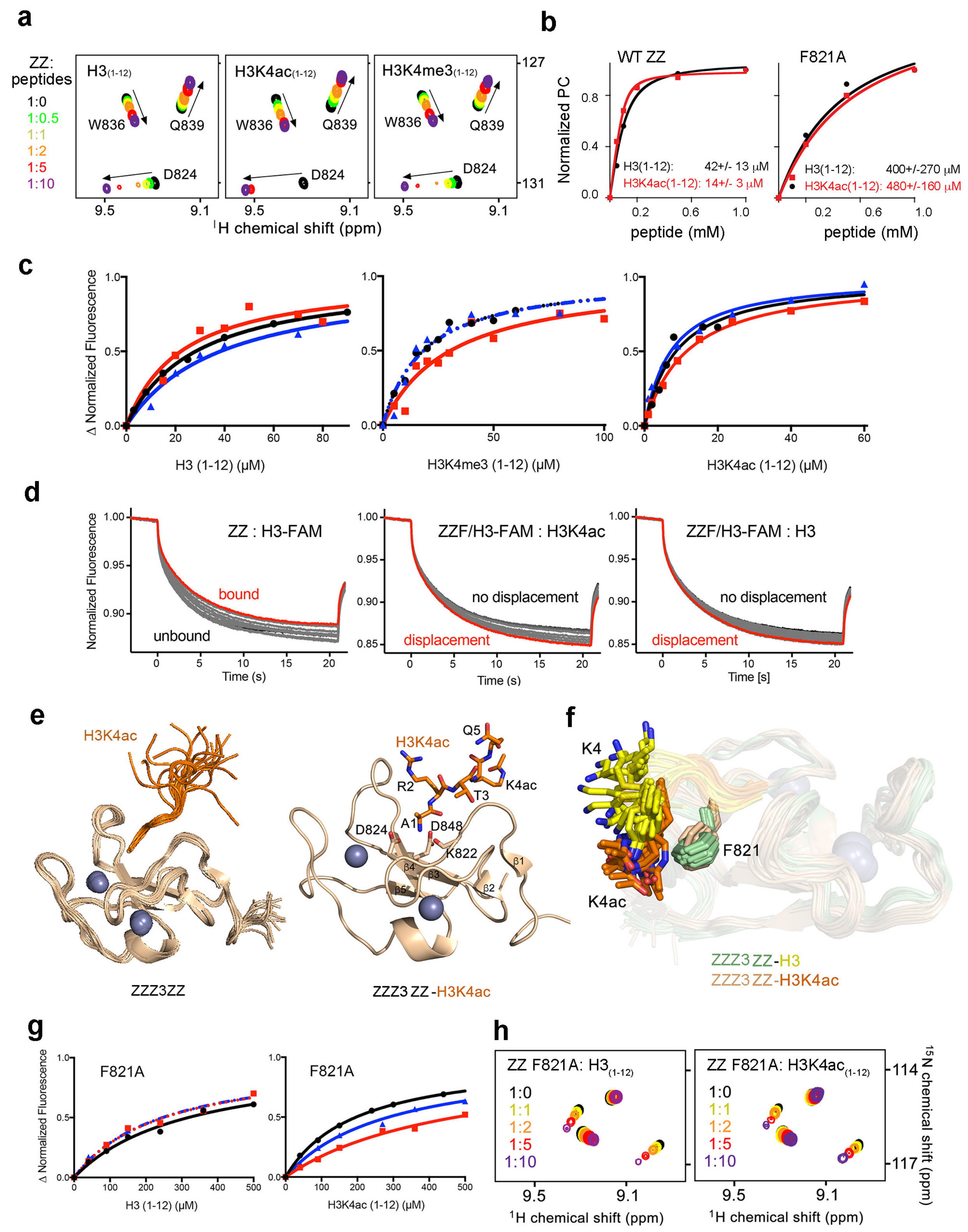

Supplementary Figure 3. Acetylation on H3K4 augments ZZ binding to histone H3.
a, Superimposed ^{1}H,^{15}N HSQC spectra of ZZZ3 $ZZ_{816\text{-}874}$ collected upon titration with unmodified H3, H3K4ac or H3K4me3 peptide (residues 1–12 of H3). Spectra are color coded

according to the protein-to-peptide molar ratio. **b,** Binding affinities of ZZZ3 $ZZ_{800\text{-}903}$ WT and F821A for indicated peptides measured by $^{1}H,^{15}N$ HSQC titration experiments. The binding isotherms were obtained using principal component analysis (PCA) implemented with TREND software. **c,** Binding curves used to determine binding affinities of ZZZ3 $ZZ_{800\text{-}903}$ to the H3 (1-12), H3K4me3 and H3K4ac peptides by fluorescence spectroscopy. **d,** Binding affinities of ZZZ3 $ZZ_{800\text{-}903}$ to the H3 (1-12) and H3K4ac peptides by MST binding assays. **e**, Left: superimposition of 20 final NMR structures. Right: Ribbon diagram of the ZZZ3 $ZZ_{816\text{-}874}$ domain (wheat) in complex with the histone H3K4ac (1-8) peptide (orange). **f**, Superimposition of the ensemble of 20 final NMR structures of H3K4(yellow)-bound ZZZ3 ZZ with the ensemble of 20 final NMR structures of H3K4ac(orange)-bound ZZZ3 ZZ. **g**, Binding curves used to determine binding affinities of ZZZ3 $ZZ_{800\text{-}903}$ F821A to the H3 (1-12) and H3K4ac peptides by fluorescence spectroscopy. **h,** Superimposed $^{1}H,^{15}N$ HSQC spectra of the ZZZ3 $ZZ_{800\text{-}903}$ F821A mutant titrated with the H3 (1-12) and H3K4ac (1-12) peptides. Spectra are color coded according to the protein-to-peptide molar ratio.

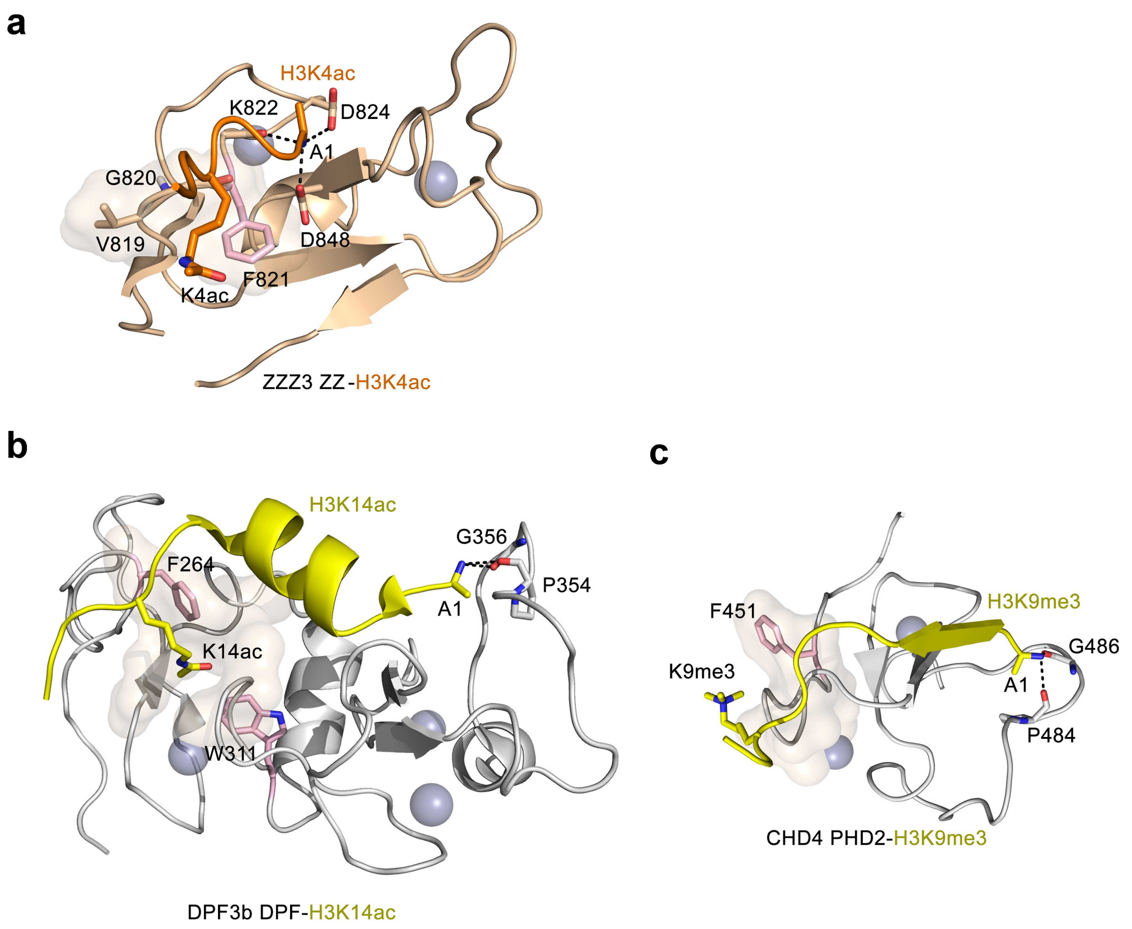

Supplementary Figure 4. Structures of the ZZZ3 ZZ (a), DPF3b DPF (b) and CHD4 PHD2 (c) readers in complex with the indicated histone peptides. The aromatic residues are colored pink and the histone peptides in orange (a) or yellow (b, c). Surfaces of the domains within 5Å of the modified lysine residues are shown in wheat. PDB IDs: 5I3L (b) and 2L75 (c).

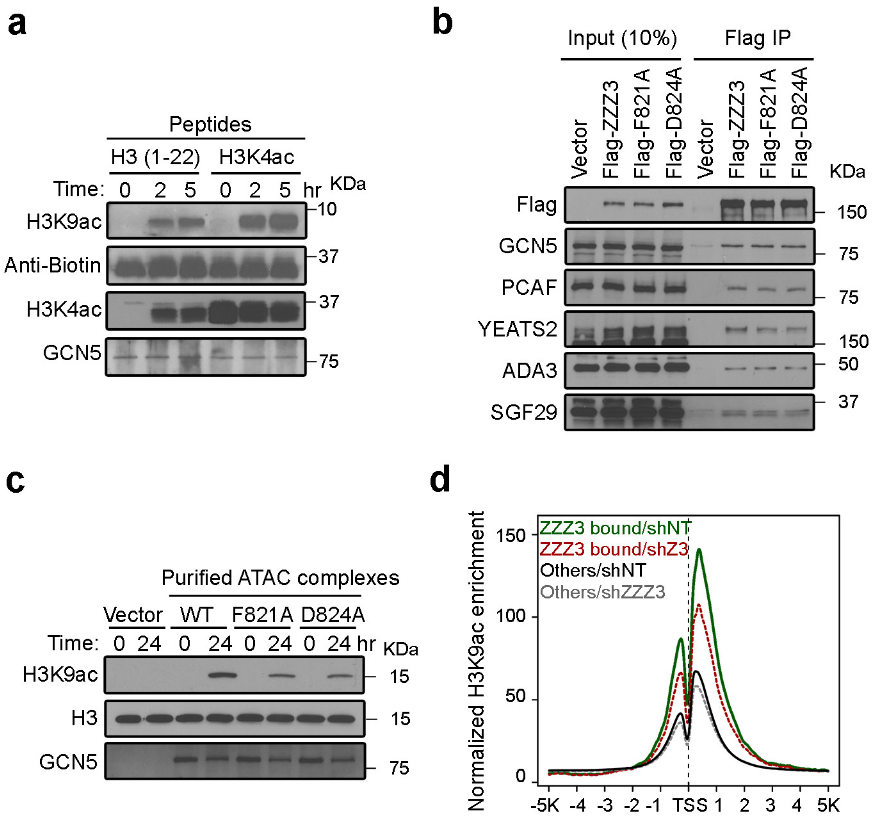

Supplementary Figure 5. Recognition of H3 by ZZZ3 is critical for ATAC-dependent histone acetylation. **a**, The ATAC complex shows a higher HAT activity on the Lys4-acetylated H3 peptide than the unmodified H3 peptide. Western blot analysis of HAT assays of purified ATAC complexes using H3 (1-22) peptide as substrate. Biotinylated peptides are shown as loading controls. **b**, Western blot analysis of co-IP using the M2 anti-Flag antibody in stable cells expressing Flag-ZZZ3 and indicated mutants. **c,** Point mutations of ZZ domain attenuate the HAT activity of the ATAC complex *in vitro*. Western blot analysis of HAT assays of purified ATAC complexes containing WT or mutant ZZZ3 using mononucleosomes as substrate. Histone H3 is shown as a loading control. **d,** Average genome-wide H3K9ac occupancy on the promoter (5kb +/- TSS) of the ZZZ3-bound genes or non-ZZZ3 bound genes (others) in control (shNT) and ZZZ3 KD (shZZZ3).

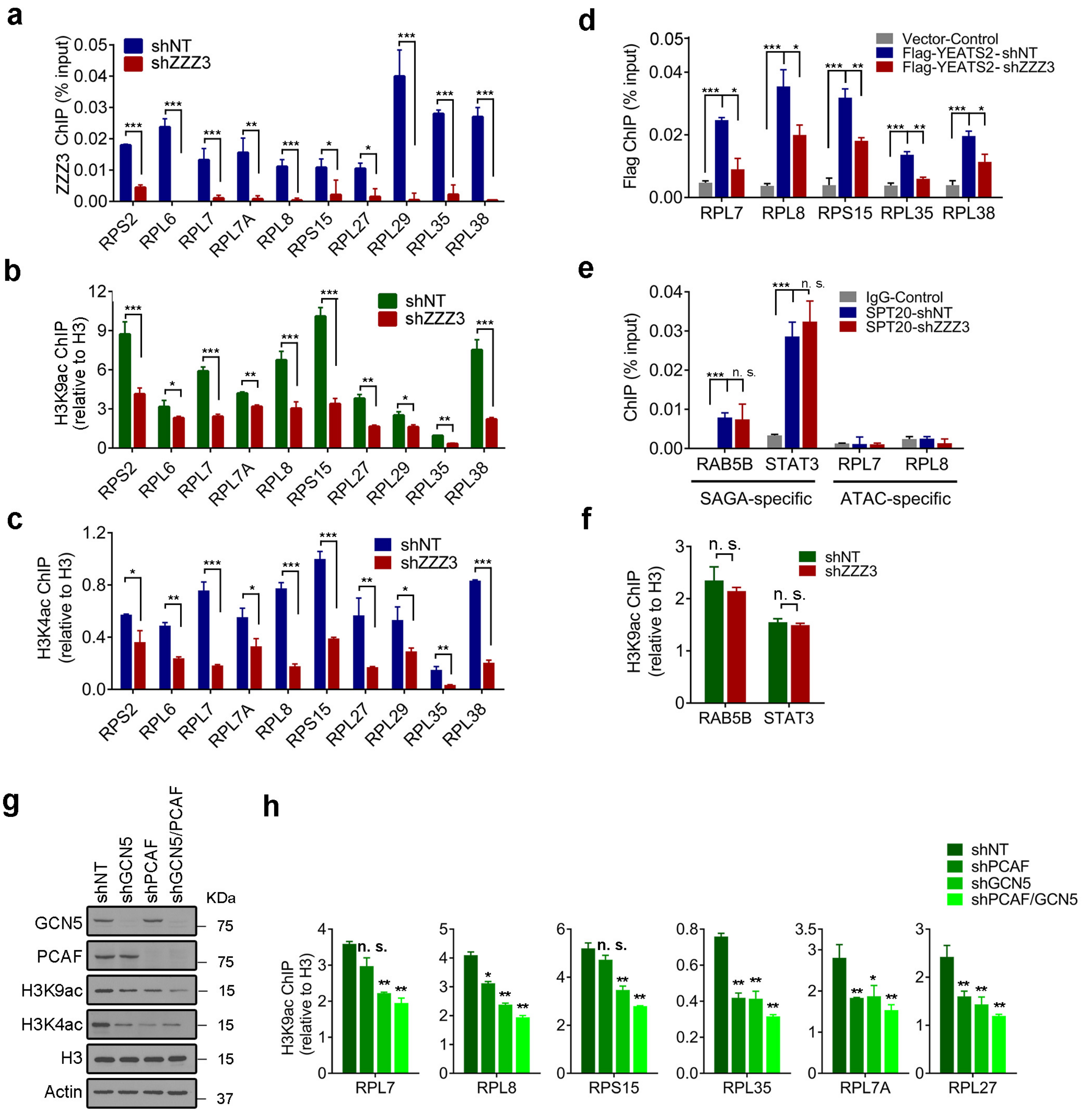

Supplementary Figure 6. ZZZ3 is required for chromatin occupancy of the ATAC complex and histone acetylation on ATAC target genes. **a-c,** qPCR analysis of ZZZ3 (a), H3K9ac (b) and H3K4ac (c) ChIP at promoters of the ribosomal protein genes in control (shNT) and ZZZ3 KD (shZZZ3) cells. **d,** qPCR analysis of Flag ChIP at promoters of the indicated ribosomal protein genes in control and Flag-YEATS2 stable cells expressing shNT or shZZZ3 shRNAs. **e,** qPCR analysis of IgG or SPT20 ChIP at promoters of the indicated genes in cells as in (a). **f,** qPCR analysis of H3K9ac at promoters of the indicated SAGA target genes in cells as in (a). **g,** Western blot analysis of global H3K9ac level in GCN5 KD (shGCN5), PCAF KD (shPCAF) and both KD (shGCN5/PCAF) cells. H3 and actin were used as loading controls. **h,** qPCR analysis of H3K9ac ChIP at promoters of indicated ribosomal protein genes in cells as in (g). Error bars indicate s.e.m. of three replicates. In all panels, n. s.: not significant; *: $p < 0.05$; **: $p < 0.01$; ***: $p < 0.001$ (two-tailed unpaired Student's *t*-test).

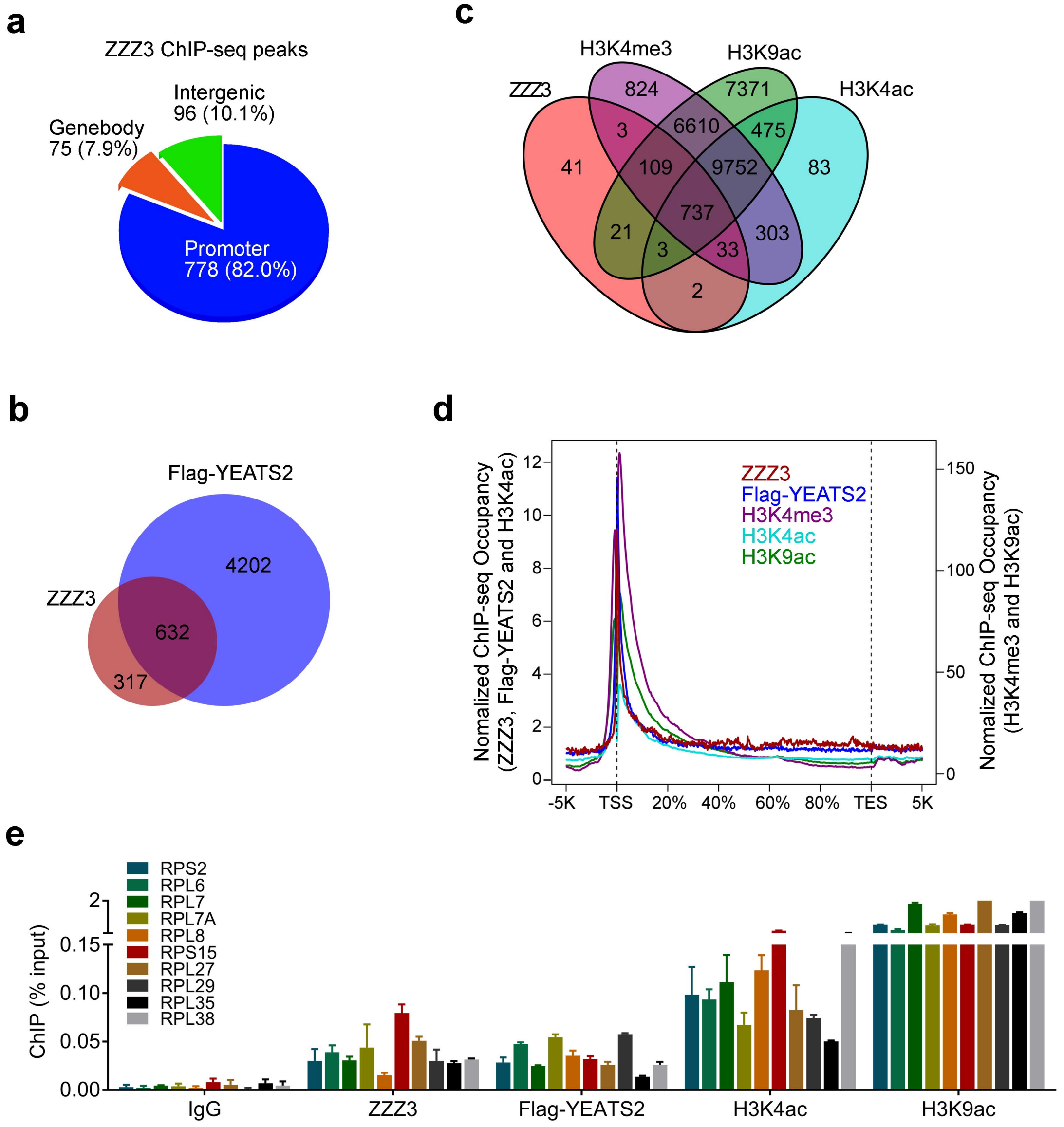

Supplementary Figure 7. ZZZ3 co-localizes with YEATS2 and active histone marks at gene promoters. a, Genomic distribution of ZZZ3 ChIP-seq peaks in H1299 cells. The peaks are enriched in the promoter regions (transcription start site (TSS) ±3kb). $p < 2.2\times10^{-16}$ (binomial test). **b**, Venn diagram showing the overlaps of ZZZ3 (red) with Flag-YEATS2 (blue). $p = 1.8\times10^{-224}$ (Hypergeometric test). **c,** Venn diagram showing the overlaps of ZZZ3 (red), H3K4me3 (purple), H3K4ac (cyan) and H3K9ac (green) occupied peaks. $p = 8.6\times10^{-231}$ (Super exact test). **d,** Average ZZZ3 (red), YEATS2 (blue), H3K4me3 (purple), H3K4ac (cyan) and H3K9ac (green) occupancies along the transcription unit. The gene body length is normalized by percentage from the TSS to transcription termination site (TES). 5 kb region upstream of TSS and 5kb region downstream of TES are also included. **e,** qPCR analysis of ZZZ3, Flag-YEATS2, H3K4ac and H3K9ac ChIP at the promoter of the representative ribosomal protein genes. IgG was used as a negative control. Error bars indicate s.e.m. of three repeats.

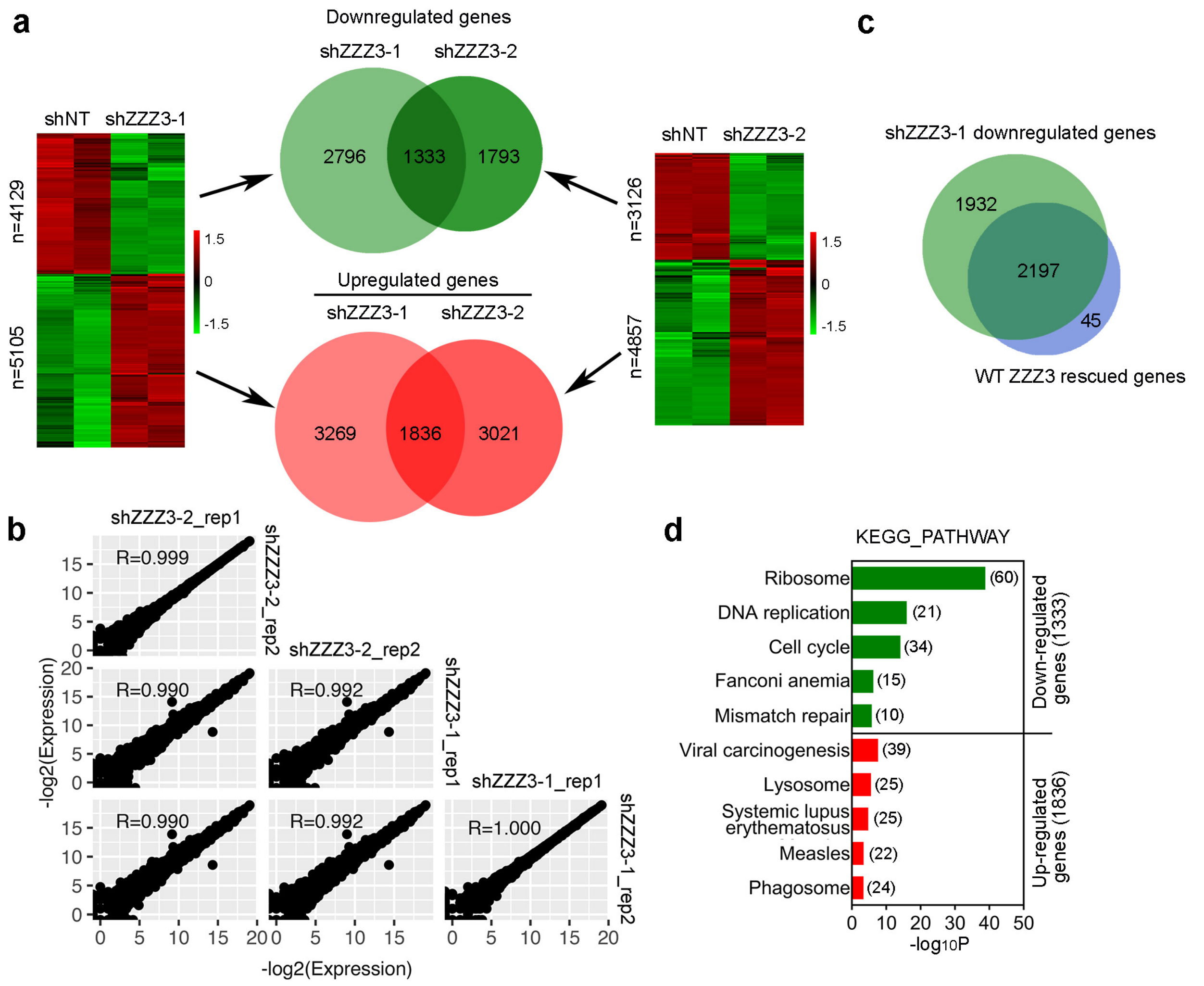

Supplementary Figure 8. ZZZ3 is required for the expression of ribosomal protein genes. **a,** Heatmaps and Venn diagrams showing significant overlap of downregulated or upregulated genes between two ZZZ3 shRNAs. Heatmaps of differentially expressed genes in control (shNT) and ZZZ3 KD cells from two biological replicates of RNA-seq experiments are shown. Fisher's exact test was used to define differentially expressed genes ($q < 0.01$). The color key represents normalized Log2 expression values. The overlap $p < 2.2 \times 10^{-16}$ (Super exact test).
b, Scatterplot for Spearman's rank correlation coefficients comparing genome-wide gene expression levels for biological replicates of RNA-seq samples using two ZZZ3 shRNAs. p-values $<2.2 \times 10^{-16}$ for all pairwise comparisons. **c,** Venn diagram showing overlap of down-regulated genes in shZZZ3-1 KD cells and genes rescued by WT ZZZ3. **d,** KEGG pathway analysis of down- (green) or up-regulated (red) genes in ZZZ3 KD compared to control cells. The numbers of genes within each functional pathway are shown in parenthesis. Fisher's exact test was used to identify the biological function with significant P-values (Benjamini–Hochberg corrected $p < 0.05$).

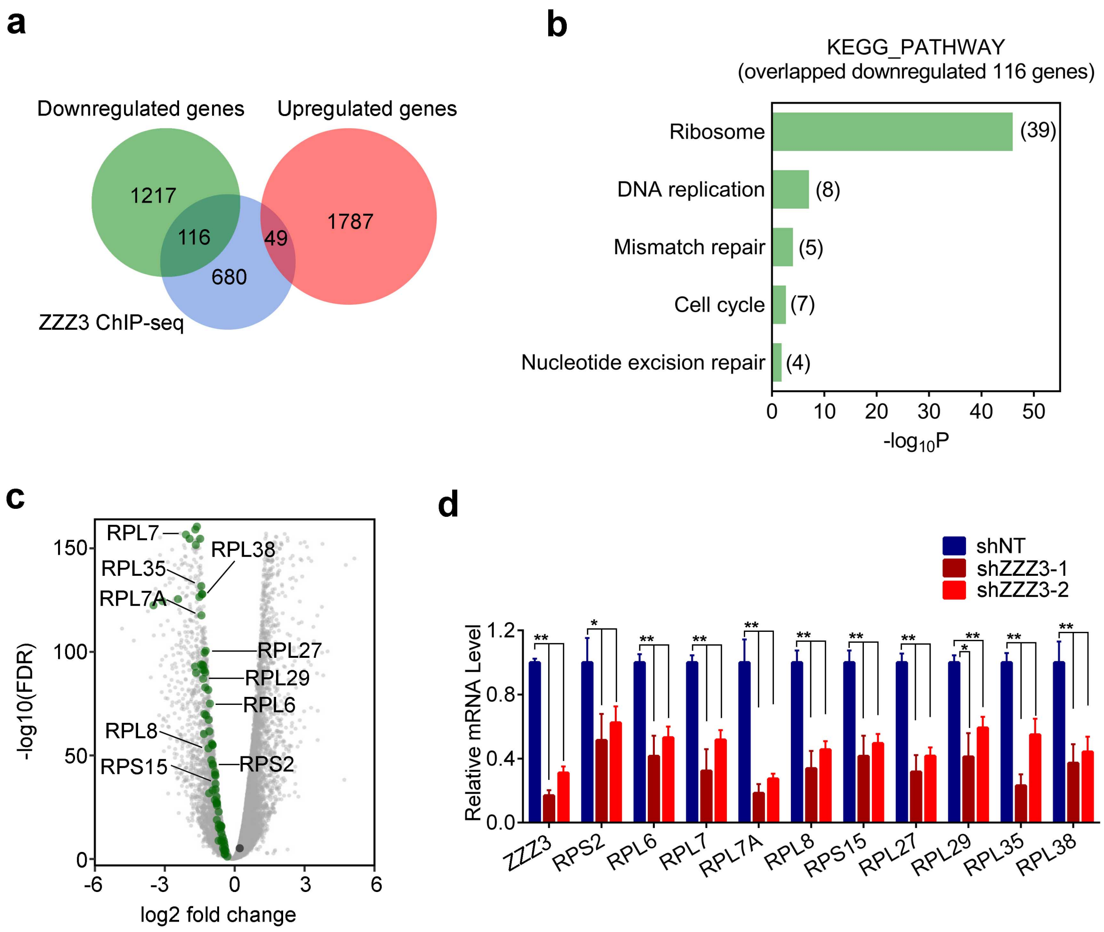

Supplementary Figure 9. ZZZ3 is required for the expression of ribosomal protein genes. **a,** Venn diagram showing the overlaps of ZZZ3 occupied genes (blue) with downregulated (green) or upregulated genes (red) upon ZZZ3 KD. $p = 1.0 \times 10^{-20}$ for overlapped downregulated genes and p=0.96 for overlapped upregulated genes (Hypergeometric test). **b,** KEGG pathway analysis of down-regulated ZZZ3-occupied genes. The numbers of genes within each functional pathway are shown in parenthesis. Fisher's exact test was used to identify the biological function with significant P-values (Benjamini–Hochberg corrected $p < 0.05$). **c,** Volcano plot of differentially expressed genes in ZZZ3 KD cells compared with control cells. 60 down-regulated ribosomal protein genes are shown in green dots and the 10 representative ribosomal protein genes are labeled. FDR, false discovery rate. **d,** Quantitative RT-PCR (qRT-PCR) analysis of the expression of the randomly selected ribosomal protein genes in control (shNT) and ZZZ3 KD (shZZZ3) cells. Error bars indicate s.e.m. of three replicates. *: $p < 0.05$; **: $p < 0.01$; (two-tailed unpaired Student's t-test).

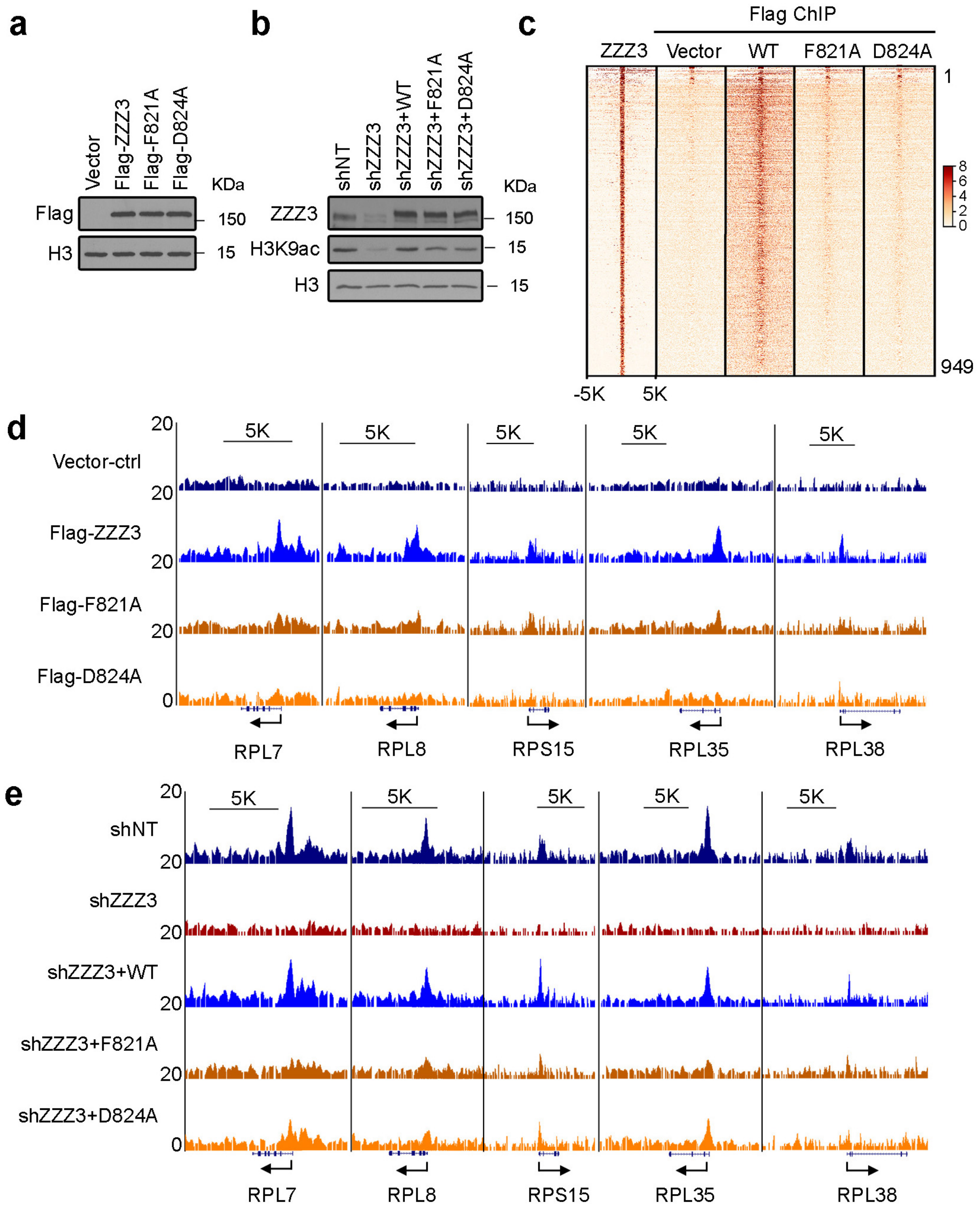

Supplementary Figure 10. ZZ domain is required for ZZZ3 chromatin recruitment.
a, Western blot analysis of ZZZ3 protein levels in H1299 cells stably expressing Flag-tagged WT ZZZ3 or the indicated mutants. H3 was used as a loading control. **b,** Western blot analysis of ZZZ3 and H3K9ac levels in control (shNT), ZZZ3 KD cells, and KD cells stably expressing shRNA-resistant WT ZZZ3 or the indicated mutants. H3 was used as a loading control. **c,** Heatmap of the normalized Flag ChIP-seq signal densities in Vector, WT and indicated mutants' stable cells. The signal is centered on ZZZ3 binding site in a ± 5kb window. **d,** Representative genome-browser views of Flag ChIP-seq signals in Vector, WT and indicated mutants' stable cells. TSS is indicated by an arrow. **e,** Representative genome-browser views of ZZZ3 ChIP-seq signals in cells as in (b). TSS is indicated by an arrow.

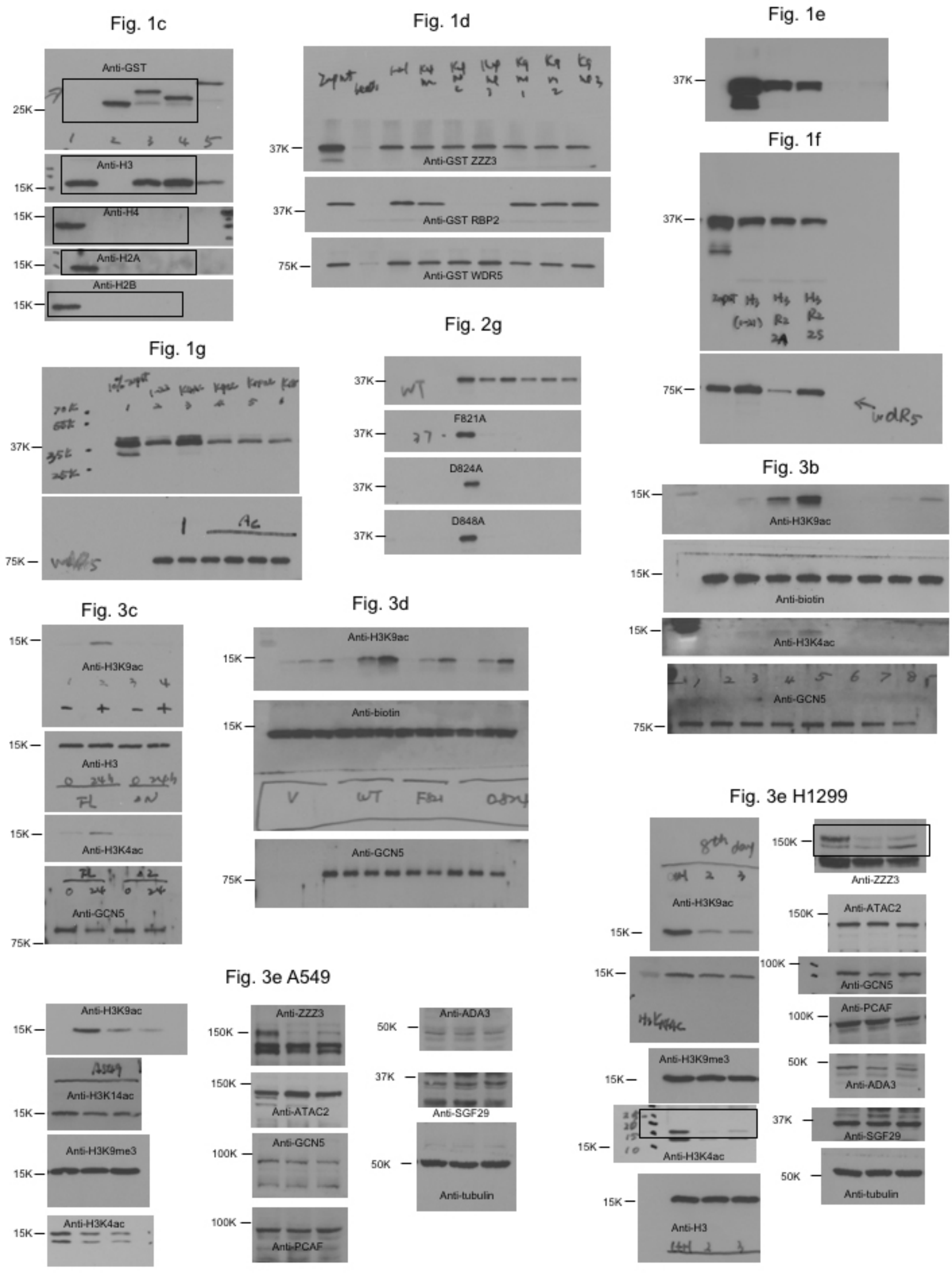

Supplementary Figure 11. Uncropped Western blots.

Supplementary Table 1. NMR and refinement statistics for the ZZ-H3 and ZZ-H3K4ac structures

	**ZZ-H3**	**ZZ-H3K4ac**
**NMR distance and dihedral constraints**		
Distance constraints		
Total NOE	2357	2058
Intra-residue	651	618
Inter-residue	1706	1440
Sequential ($\|i - j\| = 1$)	603	479
Medium-range ($\|i - j\| < 4$)	646	607
Long-range ($\|i - j\| > 5$)	400	306
Intermolecular	57	48
Hydrogen bonds	11	11
Total dihedral angle restraints		
ϕ	30	27
ψ	29	28
**Structure statistics**		
Violations (mean and s.d.)		
Number of dihedral angle violation (> 5º)	1	0
Number of distance constraint violation (>0.3 Å)	1	0
Max. dihedral angle violation (º)	11.7	2.8
Max. distance constraint violation (Å)	0.342	0.278
Deviations from idealized geometry		
Bond lengths (Å)	0.0108 ± 0.001	0.0117 ± 0.001
Bond angles (º)	2.633 ± 0.025	2.780 ± 0.056
Average pairwise r.m.s. deviation** (Å)		
Heavy	1.254 ± 0.114	0.755 ± 0.098
Backbone	0.700 ± 0.105	0.351 ± 0.083

**Pairwise r.m.s. deviation to lowest energy structure was calculated among 20 refined structures. Residues 816-874 of ZZZ3 and 1-4 of H3 were used.

Supplementary Data 1. Lists of ChIP-seq peaks and occupied genes of ZZZ3, YEATS2, H3K4ac, H4K9ac in H1299 cells; lists of Flag-ChIP-seq peaks in H1299 cells stably expressing Flag-ZZZ3 (WT), Flag-ZZZ3-F821A, Flag-ZZZ3-D824A or the vector control; lists of ZZZ3 ChIP-seq peaks in control, ZZZ3 KD, and ZZZ3 KD cells expressing ZZZ3 (WT), ZZZ3-F821A, or ZZZ3-D824A.

Supplementary Data 2. List of down- or up-regulated genes in shZZZ3-1 or shZZZ3-2 shRNA treated H1299 cells. Overlaps with ZZZ3 ChIP-seq occupied genes and KEGG analysis are also included.

Supplementary Data 3. List of oligoes used in qRT-PCR and ChIP-qPCR analysis.
