# Supplementary material for: The ZZ-type zinc finger of ZZZ3 modulates the ATAC complex-mediated histone acetylation and gene activation
Source: Nat Commun. 2018 Sep 14;9:3759. doi: 10.1038/s41467-018-06247-5 (PMC6138639; doi:10.1038/s41467-018-06247-5)
Supplement: Supplementary file 3 — Description of Additional Supplementary Files [file 41467_2018_6247_MOESM3_ESM.pdf]

Description of Additional Supplementary Files

File Name: Supplementary Data 1
Description: Lists of ChIP-seq peaks and occupied genes of ZZZ3, YEATS2, H3K4ac, H4K9ac in H1299 cells; lists of Flag-ChIP-seq peaks in H1299 cells stably expressing Flag-ZZZ3 (WT), Flag-ZZZ3-F821A, Flag-ZZZ3-D824A or the vector control; lists of ZZZ3 ChIP-seq peaks in control, ZZZ3 KD, and ZZZ3 KD cells expressing ZZZ3 (WT), ZZZ3-F821A, or ZZZ3-D824A.

File Name: Supplementary Data 2
Description: List of down- or up-regulated genes in shZZZ3-1 or shZZZ3-2 shRNA treated H1299 cells. Overlaps with ZZZ3 ChIP-seq occupied genes and KEGG analysis are also included

File Name: Supplementary Data 3
Description: List of oligoes used in qRT-PCR and ChIP-qPCR analysis
